# Ohio State University

**Published:** 1895-11

**Authors:** 


### OHIO STATE UNIVERSITY.

IN our July number we very regretfully announced that the veterinary department of the above university was to be closed. We are informed by President Canfield that such is not to be the case, but that it is to be placed on the same basis as it was prior to being made a school with a full corps of veterinary instructors. As a department of the university it will now be presided over by Professor D. S. White, formerly the third instructor in the school. Associated with him will be Dr. H. M. Ball, of Columbus, Ohio, a graduate of the American Veterinary College, class of 1888. We were informed that Professor Paul Fischer, formerly one of the chief instructors, now connected with the Utah State University, was to return again, but we find this announcement was premature. Professor Detmers will not be connected with the institution. As the department will have but two veterinarians on the teaching staff, of course it cannot be recognized as a college by any of the leading veterinary associations of the country. By these changes the expense of running the department have been materially lessened, which are given as the chief reasons for the changes. We believe that Dr. Ball has had no prior experience as an instructor. We make these corrections at the request of President Canfield.

